# Successful Conservative Management of a Patient With Symptomatic Cervical Perineural Cysts: A Case Report

**DOI:** 10.7759/cureus.59902

**Published:** 2024-05-08

**Authors:** Stavros Stamiris, Christos Karampalis, Dimitrios Stamiris, Elissavet Anestiadou, Pavlos Christodoulou

**Affiliations:** 1 Department of Orthopaedics, 424 General Military Hospital, Thessaloniki, GRC; 2 4th Surgical Department, Papanikolaou General Hospital of Thessaloniki, Thessaloniki, GRC

**Keywords:** clinical case report, cervical spine disorders, cervical radiculopathy, perineural cysts, tarlov cysts

## Abstract

Perineural cysts, also known as Tarlov cysts, are rare benign cerebrospinal fluid-filled cysts usually located at the junction of the posterior nerve root and the dorsal root ganglion and are usually asymptomatic. They are most commonly found in the sacral region and are uncommon in the cervical spine. Despite their rarity, symptomatic cases may present with neurological symptoms due to the compression of adjacent neurological structures. Symptomatic cervical perineural cysts are extremely rare, and there is limited consensus on management strategies. We present the case of a 56-year-old woman who presented with a four-week history of radicular symptoms involving the right C7 and C8 nerve roots, including neck and arm pain, paresthesias, and mild triceps weakness. Magnetic resonance imaging revealed two perineural cysts at the C6-C7 and C7-T1 levels. A conservative approach was chosen with a 14-day course of oral corticosteroids, use of a soft collar, and activity restrictions. Following this conservative treatment, a significant reduction in symptoms and complete neurological recovery were achieved. This case highlights the efficacy of conservative approaches in selected cases of mildly symptomatic cervical perineural cysts and contributes to a better understanding of management strategies for this condition.

## Introduction

Perineural cysts, also known as Tarlov cysts, are rare encapsulated cerebrospinal fluid (CSF)-filled sacs that are formed in the interval between the perineurium (the connective tissue sheath surrounding a bundle of nerve fascicles) and endoneurium (connective tissue surrounding individual nerve fibers) of nerve roots and are usually located at the junction of the posterior nerve root and the dorsal root ganglion [1]. Primarily, they are found in the sacral spine and on rare occasions have been reported in higher spinal levels, more prevalent in the cervical spine [2,3]. The prevalence of perineural cysts in the lumbosacral region is estimated at 4.6%, but their occurrence in the cervical spine remains poorly understood and their incidence has not been studied to date [1,2]. Few documented cases exist, possibly due to the asymptomatic nature of most cysts, often discovered incidentally during radiologic examinations for unrelated conditions.

Although often asymptomatic, symptomatic cases can present with neurological symptoms due to the compression of adjacent neural structures. Symptomatic cervical perineural cysts are exceedingly rare, with limited consensus on treatment strategies [1]. Due to their rarity, these cysts still pose therapeutic dilemmas, and there is limited consensus on management strategies to guide clinicians. Management of symptomatic perineural cysts varies, encompassing conservative approaches such as non-steroidal anti-inflammatory drugs (NSAIDs), oral steroids, transforaminal epidural injections, percutaneous aspiration, and surgical interventions [4]. Diagnostic imaging, particularly magnetic resonance imaging (MRI), plays a crucial role in assessing cyst characteristics and planning appropriate treatment modalities.

In this article, we report a unique case of two symptomatic cervical perineural cysts in a 56-year-old female patient, who was treated successfully conservatively. We believe that this case report is of value as the symptomatic cervical Tarlov cyst is under-reported in the literature and will help in establishing a treatment algorithm. This case report is presented in accordance with CAse REport (CARE) guidelines [5]. The patient has been fully informed, and written informed consent has been obtained for the publication of this case report and accompanying images.

## Case presentation

A 56-year-old woman presented at the outpatient department of a tertiary care military hospital with longstanding pain in the posterior neck and right upper extremity. She reported that the pain had first appeared two years ago without any precipitating event and had continued since then with fluctuations. Over the past month, she reported that the symptoms had worsened and became persistent, graded by the patient as 8/10 on the Visual Analogue Scale (VAS). She also reported the development of a burning sensation and numbness in her index, middle, ring, and little fingers over the last two weeks. She had already been treated with NSAIDs (diclofenac 75 mg twice daily) prescribed by a general practitioner for 10 days without any improvement in her symptoms.

On clinical examination, the patient had a normal neck range of motion and negative Spurling's test on the right side, but the symptoms of radicular pain and paresthesia were aggravated by tilting her head in the opposite direction with simultaneous extension of the contralateral arm. Upper extremity deep tendon reflexes (biceps, brachioradialis, triceps) were normal. On neurological examination, mild hypesthesia was present in the left ulnar forearm, hand, and index, middle, ring, and little fingers to light touch, corresponding to the dermatomal distribution of C7 and C8 roots, but not to pinprick. A subtle weakness was noted in the extension of the elbow (triceps), corresponding to C7 myotomal distribution, graded 4+/5 on the Medical Research Council (MRC) muscle strength scale [6], while the other myotomes were within normal limits.

**Figure 1:**
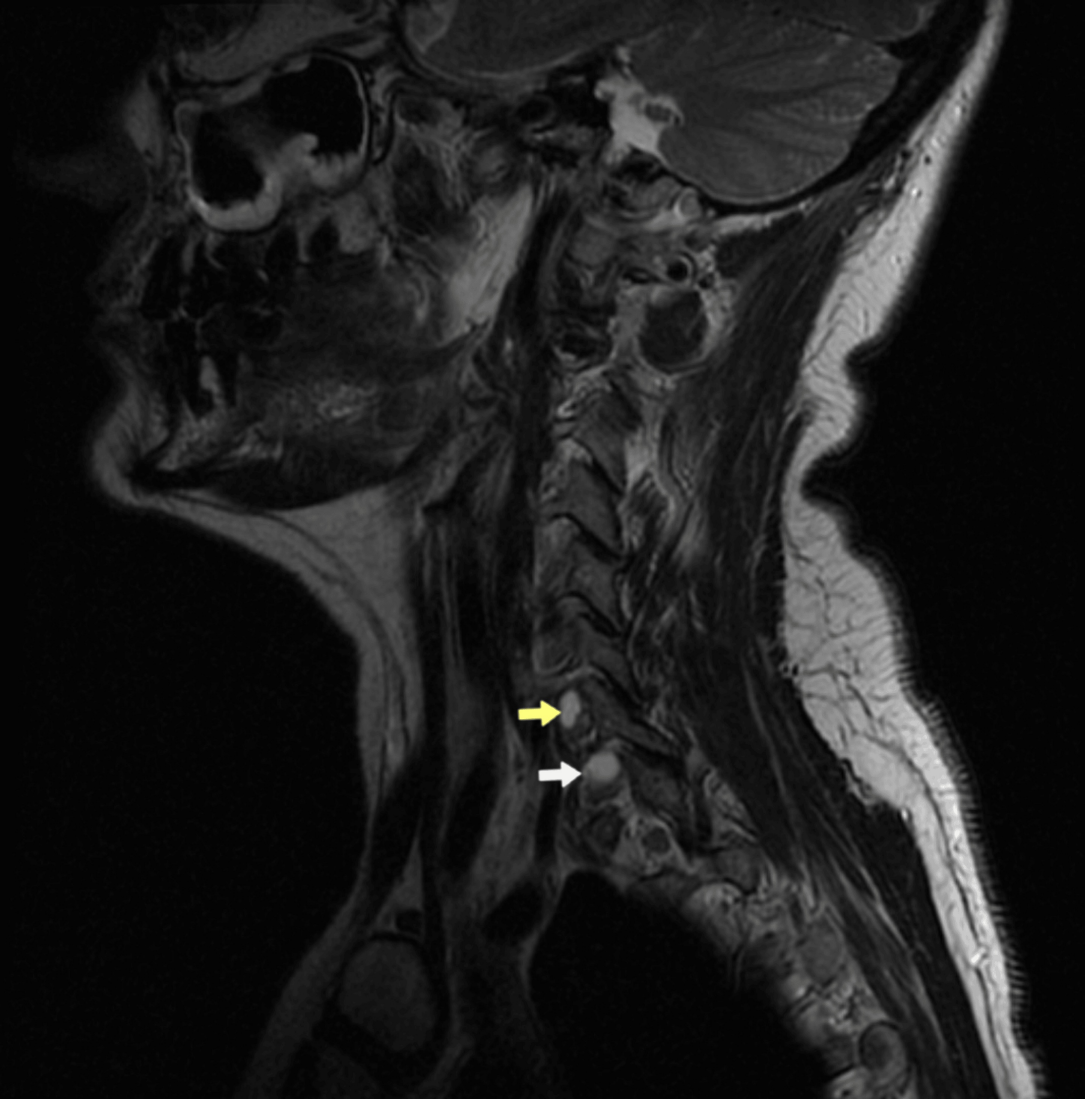
Magnetic resonance image of the cervical spine. Sagittal T2-weighted image showing two Tarlov cysts in the right foramen at levels C6-C7 (yellow arrow) and C7-T1 (white arrow), resulting in the compression of the C7 and C8 nerve roots, respectively, explaining the presence of neurological symptoms in two distinct dermatomes.

**Figure 2:**
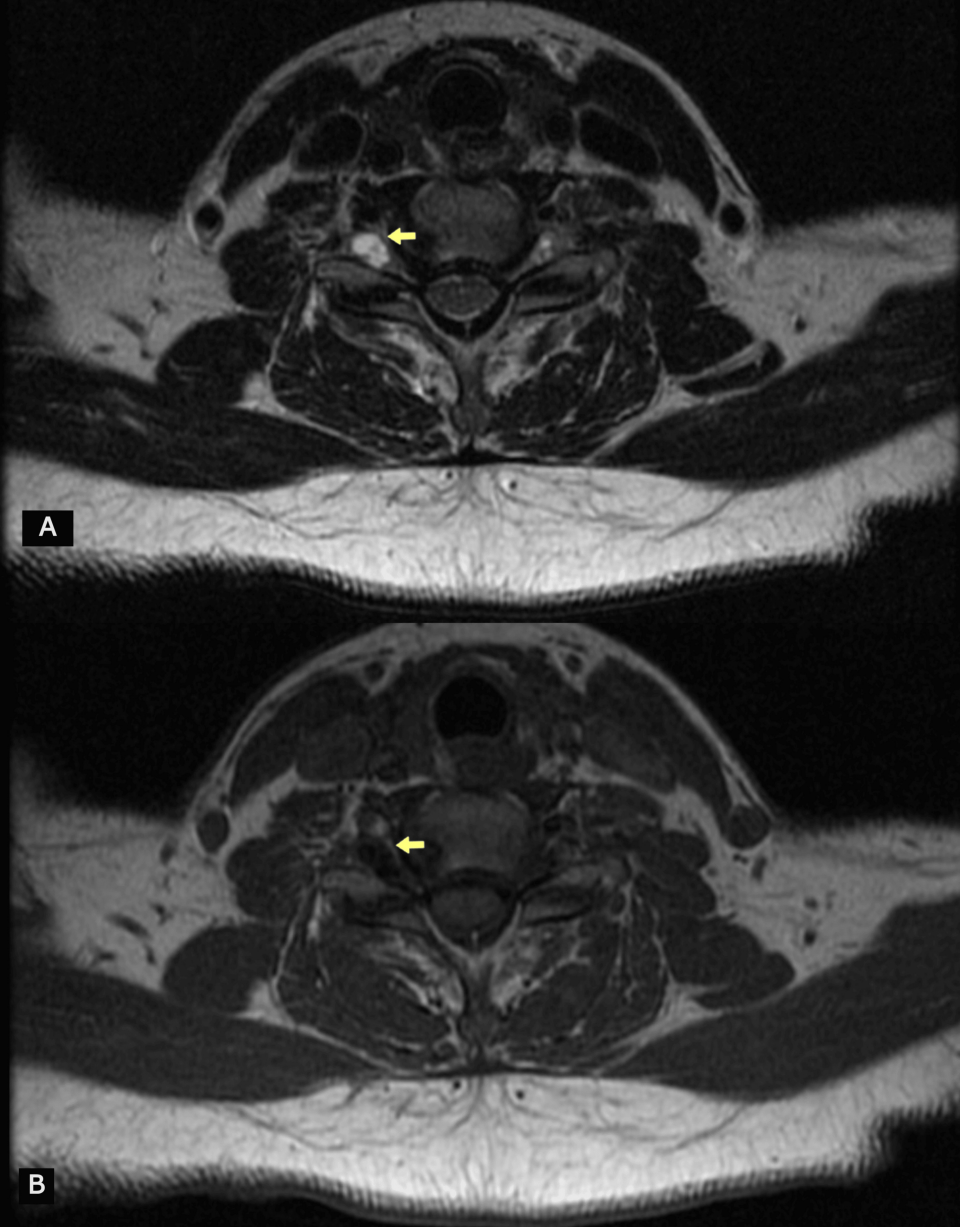
Magnetic resonance image of the cervical spine. Axial images (T2-weighted (A) and T1-weighted (B)) revealing the presence of a perineural cyst at the right neural foramen formed by C6 and C7 vertebra. The perineural cyst (arrow) is hypointense on the T1-weighted image and hyperintense on the T2-weighted image.

**Figure 3:**
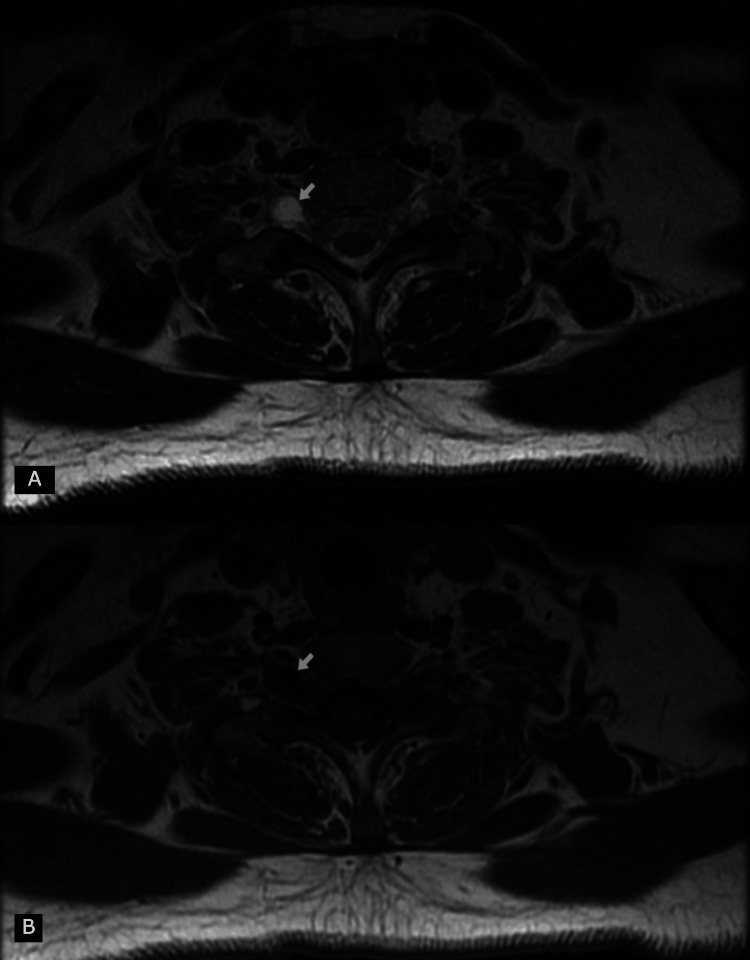
Magnetic resonance image of the cervical spine. Axial images (T2-weighted (A) and T1-weighted (B)) revealing the presence of a perineural cyst at the right neural foramen formed by C7 and T1 vertebra. The perineural cyst (arrow) is hypointense on the T1-weighted image and hyperintense on the T2-weighted image.

An MRI scan of the cervical spine revealed the presence of two perineural cysts at the right neural foramens at levels C6-C7 and C7-T1, explaining the presence of neurological symptoms in two distinct dermatomes (Figure 1). In the axial plane, the cysts measured 8.34×5.03 mm at the C6-C7 level (Figure 2) and 8.54×7.19 mm at the C7-T1 level (Figure 3), respectively. The cysts were hyperintense on the T2-weighted image and hypointense on the T1-weighted image.

Subsequent electrodiagnostic evaluation was ordered and revealed normal motor and sensory conduction velocities but reduced recruitment of the triceps and extensor digitorum communis with no automatic activity, possibly indicating acute C7 root damage.

Based on these findings, the patient was treated for C7-C8 radiculopathy. Due to the presence of neurological manifestations and the failure of previous NSAID's treatment, she was prescribed a 14-day oral corticosteroid regimen (prednisolone, initially 20 mg twice daily for five days followed by a gradual taper over a period of nine days). Additionally, she was instructed to wear a soft cervical collar and adhere to activity restrictions, which included avoiding extreme stretching of the arm and neck.

On re-evaluation after two weeks of conservative therapy, the patient showed a marked reduction in radicular symptoms and complete recovery of her neurological manifestations. At the last follow-up at six months, she had a satisfactory outcome with over 90% improvement in her symptoms. At that time, a repeat MRI was suggested to monitor the status of the perineural cysts. However, the patient declined this suggestion as she was satisfied with the outcome of her treatment and expressed no desire for further investigation.

## Discussion

Perineural cysts arise within the perineural space between the endoneurium (which is derived from the pia mater) and the perineurium (formed by the arachnoid mater). They develop along nerve roots, either at or distal to the junction of the dorsal root and the dorsal ganglion [7]. They present as single or multilevel cysts, mainly at the sacral level, but have also been reported to exist at other spinal levels, particularly the cervical level. The prevalence of Tarlov cysts in the sacral region in the general population ranges from 1.5% to 13.2% and appears to be more common in women [8].

Several theories have been proposed for the pathogenesis of perineural cysts, including inflammation, trauma, congenital origin, or degenerative process. Regardless of the origin, the currently accepted mechanism of cyst development is a disruption of the CSF-venous drainage at the perineurial-epineurial junction. Enlargement of the cyst occurs due to microcommunication between the cyst and the subarachnoid space and a valve-like mechanism that allows CSF influx and restricts CSF reflux, leading to the expansion of the cyst [8].

Perineural cysts are usually incidental findings without clinical significance. However, in some cases, they may produce symptoms. When symptomatic, cervical perineural cysts usually present with radicular pain as the main symptom that is experienced by the patient as a cramping, burning, or tingling sensation in a non-specific dermatomal distribution [1,4]. The symptoms are usually aggravated when the patients tilt their heads in the opposite direction or when in a supine position [2,4,9]. More severe cases can be presented with muscle strength or sensory deficit in a specific myotomal or dermatomal pattern [9,10]. Our patient presented with both radicular symptoms and mild neurological manifestations including hypesthesia in C7-C8 dermatomal distribution and C7 myotomal distribution.

MRI remains the method of choice for the diagnosis of perineural cysts. The cystic lesions appear in close proximity to the dorsal root ganglion and have a hypointense signal on T1-weighted imaging and hyperintense signal on T2-weighted imaging, similar to the CSF [11].

There is no consensus on the treatment algorithm for patients with symptomatic cervical perineural cysts. Aristeidis et al. described a successful conservative approach with oral NSAIDs, soft cervical collar, and activity modification for the treatment of a symptomatic perineural cyst with only radicular symptoms [4]. Jain et al. and Dharmadhikari and Gokhale reported the successful treatment of patients with radicular pain and sensory/motor deficits by employing oral steroid medication [9,12]. Several authors have also described successful relief in patients with symptomatic cervical perineural cysts and the presence of neurological symptoms after transforaminal epidural steroid injections (TFESI) [1,2]. Lastly, Nathani et al. reported the successful employment of physiotherapy in a patient with a cervical perineural cyst associated with cervical radiculopathy, adding it as an adjunct therapeutic option for the conservative treatment of these patients [13]. The aforementioned conservative treatment options suffice for most cases of symptomatic cervical perineural cysts with mild or no neurological symptoms.

Surgical treatment is under-reported in the literature for cervical perineural cysts and is limited in refractory cases to the conservative treatment, due to the technical difficulties and challenges. The repair of such small lesions can be associated with complications of nerve root injury, postoperative CSF leakage, and subsequent persistent neuropathic pain. Surgical innervations for symptomatic perineural cysts include decompressive laminectomy, cyst cauterization, fenestration and imbrication, cyst excision, cyst shunting, microsurgical cyst resection, and neck ligation together with duraplasty or plication of the cyst wall [14,15]. Kameda et al. performed a meta-analysis on the efficacy of surgical treatment options in symptomatic perineural cysts. He reported an efficacy of 81% in the resolution of symptoms, a complication rate of 16.9%, and a reoperation rate of 6.7% [14]. Feigenbaum et al. reported the results from a prospective cohort study of 37 patients with symptomatic cervical perineural cysts treated with surgical intervention. All the patients initially underwent selective nerve root block but had only temporary relief of symptoms. Surgical treatment included posterior cervical foraminotomy and cyst decompression. The outcome of interest was the presence of postoperative improvement in the 36-Item Short Form Health Survey (SF-36). Patients reported improvement in all subcategories of the SF-36 survey, with a cut-off of 4/8 being statistically significant [16].

A less invasive treatment option for symptomatic patients is percutaneous aspiration of the cyst under fluoroscopic guidance. Although effective in treating symptoms, it appears to be associated with a high recurrence rate [17]. For this reason, Lee et al. advocated the use of percutaneous aspiration not as a treatment option, but as a diagnostic procedure for patient selection prior to operative treatment [18]. To mitigate the high recurrence rates after aspiration, another technique has been proposed that involves percutaneous aspiration of the cyst, followed by injection of a fibrin sealant. Murphy et al. in a retrospective cohort study reported a recurrence rate of 10.8% [19].

Murphy et al. performed a meta-analysis comparing surgical with percutaneous techniques for the treatment of symptomatic perineural cysts. He concluded that both treatment options provided a similar rate of symptom relief. In addition, they found that, although the surgical group had higher complication rates (21% versus 12.47% in the aspiration group), the incidence of cyst recurrence was much lower in the surgical group (8% versus 20% in the aspiration group) making operative treatment a superior treatment option in terms of long-term efficacy [15].

In our case, the patient presented with symptoms of C7-C8 radiculopathy. In line with the previous studies, due to mild neurological manifestations, we initially treated her conservatively with a short course of oral cortisone and activity modification [9,12]. Due to the sufficient recovery, no further treatment was needed. Despite the short follow-up period, this case report is of value as the symptomatic cervical Tarlov cyst is under-reported in the literature and this manuscript will help in establishing a treatment algorithm.

## Conclusions

Our report describes a rare case of symptomatic cervical perineural cysts in a 56-year-old woman. Through conservative treatment with oral corticosteroids and activity modification, we achieved significant symptomatic relief and complete neurological recovery. Our results underscore the efficacy of non-invasive management in selected cases of mild symptomatic cervical perineural cysts, contributing to the ongoing development of treatment algorithms for this rare condition.

## References

[1] Lee J, Kim K, Kim S (2018). Treatment of a symptomatic cervical perineural cyst with ultrasound-guided cervical selective nerve root block: a case report. Medicine (Baltimore).

[2] Kim K, Chun SW, Chung SG (2012). A case of symptomatic cervical perineural (Tarlov) cyst: clinical manifestation and management. Skeletal Radiol.

[3] Tokgöz MA, Kılıçaslan ÖF, Parlak AE (2021). Multiple perineural cysts in the cervical, thoracic, and lumbar vertebrae of a mature individual. Jt Dis Relat Surg.

[4] Aristeidis ZH, Apostolos FC, Dimitris AL (2015). Symptomatic cervical perineural (Tarlov) cyst: a case report. Hippokratia.

[5] Gagnier JJ, Kienle G, Altman DG, Moher D, Sox H, Riley D (2013). The CARE guidelines: consensus-based clinical case reporting guideline development. Glob Adv Health Med.

[6] (1976). Aids to the examination of the peripheral nervous system. https://www.ukri.org/wp-content/uploads/2021/12/MRC-011221-AidsToTheExaminationOfThePeripheralNervousSystem.pdf?utm_medium=email&utm_source=transaction.

[7] Tarlov IM (1970). Spinal perineurial and meningeal cysts. J Neurol Neurosurg Psychiatry.

[8] Murphy K, Nasralla M, Pron G, Almohaimede K, Schievink W (2024). Management of Tarlov cysts: an uncommon but potentially serious spinal column disease-review of the literature and experience with over 1000 referrals. Neuroradiology.

[9] Jain M, Sahu NK, Naik S, Bag ND (2018). Symptomatic Tarlov cyst in cervical spine. BMJ Case Rep.

[10] Palamar D, Misirlioglu TO, Akgun K (2018). An uncommon cause of upper limb pain: cervical perineural (Tarlov) cyst chain. Am J Phys Med Rehabil.

[11] Davis SW, Levy LM, LeBihan DJ, Rajan S, Schellinger D (1993). Sacral meningeal cysts: evaluation with MR imaging. Radiology.

[12] Dharmadhikari R, Gokhale A (2016). Symptomatic bilateral cervical perineural cyst. MOJ Orthop Rheumatol.

[13] Nathani HR, Athawale V, Ratnani G (2024). Integrative physiotherapy management of cervical radiculopathy and concurrent Tarlov cysts. Cureus.

[14] Kameda-Smith MM, Fathalla Z, Ibrahim N, Astaneh B, Farrokhyar F (2024). A systematic review of the efficacy of surgical intervention in the management of symptomatic Tarlov cysts: a meta-analysis. Br J Neurosurg.

[15] Sharma M, SirDeshpande P, Ugiliweneza B, Dietz N, Boakye M (2019). A systematic comparative outcome analysis of surgical versus percutaneous techniques in the management of symptomatic sacral perineural (Tarlov) cysts: a meta-analysis. J Neurosurg Spine.

[16] Feigenbaum F, Parks SE, Martin MP, Chapple KM (2024). Surgical intervention is associated with improved outcomes in patients with symptomatic cervical spine Tarlov cysts: results from a prospective cohort study. World Neurosurg.

[17] Paulsen RD, Call GA, Murtagh FR (1994). Prevalence and percutaneous drainage of cysts of the sacral nerve root sheath (Tarlov cysts). AJNR Am J Neuroradiol.

[18] Lee JY, Impekoven P, Stenzel W, Löhr M, Ernestus RI, Klug N (2004). CT-guided percutaneous aspiration of Tarlov cyst as a useful diagnostic procedure prior to operative intervention. Acta Neurochir (Wien).

[19] Murphy K, Oaklander AL, Elias G, Kathuria S, Long DM (2016). Treatment of 213 patients with symptomatic Tarlov cysts by CT-guided percutaneous injection of fibrin sealant. AJNR Am J Neuroradiol.

